# Influence of short implants geometry on primary stability

**DOI:** 10.4317/medoral.22378

**Published:** 2018-09-28

**Authors:** José González-Serrano, Pedro Molinero-Mourelle, Beatriz Pardal-Peláez, Luis-Miguel Sáez-Alcaide, Ricardo Ortega, Juan López-Quiles

**Affiliations:** 1 Department of Dental Clinical Specialties. School of Dentistry, Complutense University, Madrid, Spain

## Abstract

**Background:**

A correct design is needed in short implants to improve primary stability (PS) in low quality bone. This study aimed to compare PS of double thread and single thread short implants.

**Material and Methods:**

Thirty implants with single thread design (PHI/SHORT-I) and 30 implants with double thread design (PHIA/SHORT-I) (Radhex®, Inmet-Garnick S.A., Guadalajara, Spain) were placed in 30 randomly selected bovine ribs. PS was assessed in implant stability quotients (ISQ) and periotest values (PV) with Osstell™ and Periotest® devices, respectively. Computed tomographies of the ribs were taken and bone quality was evaluated in Hounsfield Units (HU) using Ez3D Plus software (Vatech Co., Korea). Only implants placed in low quality bone according to Misch and Kircos classification were selected (D3 bone: 350-850 HU; and D4 bone: 150-350 HU). Ten implants were not included in the study for being placed in D1 and D2 bone. Finally, 50 implants were selected: 17 and 9 PHI/SHORT-I in D3 and D4 bone respectively, and 15 and 9 PHIA/SHORT-I in D3 and D4 bone respectively.

**Results:**

The one-way ANOVA showed statistically significant differences in ISQ (61.35 ± 4.77 in PHI/SHORT-I and 66.43 ± 4.49 in PHIA/SHORT-I, *P*<0.005) and PV (-2.76 ± 0.8 and -4.11 ± 1.24 respectively, *P*<0.005) between two implant designs in D3 bone, and statistically significant differences in ISQ (53.44 ± 3.34 in PHI/SHORT-I and 60.56 ± 1.53 in PHIA/SHORT-I, *P*<0.0001) and PV (1.13 ± 0.95 and -2.5 ± 0.61 respectively, *P*<0.0001) between two groups in D4 bone.

**Conclusion:**

Double thread design short implants resulted to have higher PS in comparison with single thread design short implants in D3 and D4 bone.

** Key words:**ISQ, osstell, periotest, primary stability, resonance frequency analysis, short implants.

## Introduction

Bone availability is a key factor for dental implant placement without injuring anatomical structures of the jaws (1). Short implants are frequently placed in order to avoid other complex and challenging procedures such as sinus floor augmentation (2), onlay graft blocks (3), lateralization of the inferior alveolar nerve (4), or distal cantilevers (5).

According to Nisand and Renouard’s classification, implants with intrabony length ≤8mm and ≤5mm are considered short and extra-short implants, respectively (6). Short implants have demonstrated to be a predictable treatment with survival rates between 74-96% at 5 years (7) or cumulative survival rates between 84-100% up to 10 years (8). This is explained by the fact that the diameter of the implant seems to be more determinant than the length of the implant to avoid overloading the peri-implant bone (9), since the stress produced during loading is concentrated around the neck of the implant (10).

However, low quality bone is known to be the determinant factor for short implant success, as it compromises primary stability (PS) at placement (11). It is known that implants inserted in low quality bone, as found when only trabecular bone contact exist, have higher failure rates (12). For this reason, when poor bone quality is present, a variation of the implant geometry can improve PS (13). This design plays an important role in providing more bone to implant contact (BIC), especially when immediate loading is demanded (14). This BIC can be increased by implant surface, implant thread number, depth or shape (15). Thus, an appropriate design is required in short implants to improve PS in low quality bone.

As there is little evidence of articles describing PS of short implants, the aim of this study was to determine in vitro the PS of two different short implant designs in low quality bone. The hypothesis of this work is that double thread short implants can achieve greater PS in comparison to single thread short implants in low quality bone.

## Material and Methods

-Dental implants

**Figure 1:**
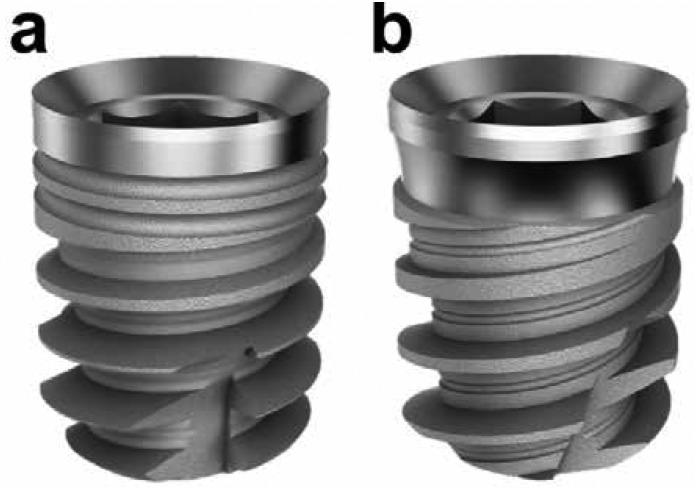
Different short implant designs used in this study: (a) tapered body and single thread design short implants (PHI/SHORT-I) and (b) tapered body and double thread design short implants (PHIA/SHORT-I).

Sixty Radhex® (Inmet-Garnick, S.A., Guadalajara, Spain) tapered short implants with a subtractive surface treatment by shot blasting were placed in thirty fresh bovine ribs. The ribs were selected randomly after the removal of the soft tissues. The implants were divided into 2 groups: 30 tapered body with single thread design implants (PHI/SHORT-I) (Fig. 1a) and 30 tapered body with double thread design implants (PHIA/SHORT-I) (Fig. 1b). Both designs were 4.5mm wide and 6mm long. Each rib received two implants, one of each type. The implants were placed by a single operator following the drilling sequence recommended by the manufacturer, with 35 Ncm or less, and using a surgical guide.

-PS measurements

PS measurements were assessed by a researcher blinded to the implant placement. A wireless resonance frequency analysis (RFA) device (Osstell AB, Gothenburg, Sweden) and a wireless electronic percussive test (Periotest M, Medizintechnik Gulden, Modautal, Germany) were used in this study.

**Figure 2:**
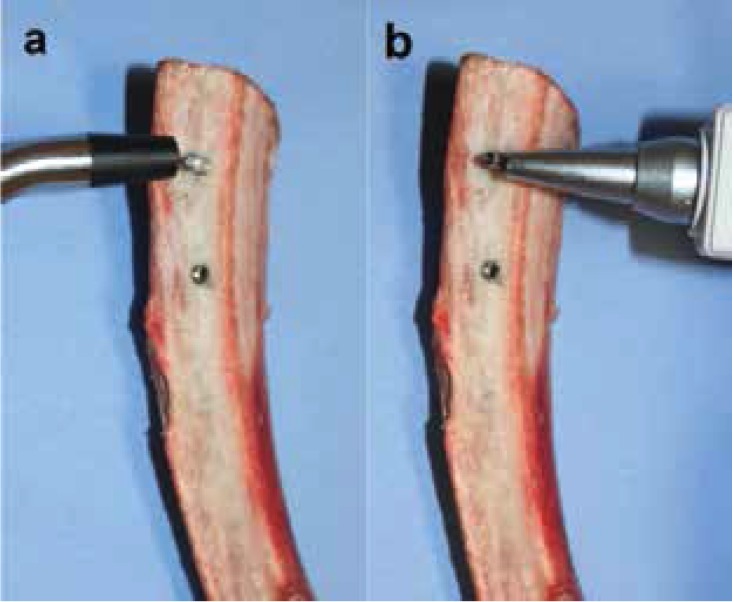
Primary stability assessment using (a) resonance frequency analysis with Osstell™ device and (b) electronic percussive test with Periotest®.

Firstly, a suitable smart-peg was inserted into the implant body, and subsequently the implant stability quotient (ISQ) was measured with the RFA device. Two perpendicular measurements were taken for each implant according to the manufacturer’s instructions (Fig. 2a). In order to eliminate measurement bias, the mean obtained was considered the value of the implant. The ISQ values ranged between 1 (minimum stability) to 100 (maximum stability). According to the manufacturer, ISQ values were classified as “high stability” (ISQ >70), “medium stability” (ISQ between 60 and 69), and “low stability” (ISQ <60).

Abutments were placed and periotest values (PV) were checked in each implant. Three consecutive measurements in different directions were taken following the manufacturer’s instructions (Fig. 2b). In order to eliminate measurement bias, the mean obtained was considered the value of the implant. The PV ranged between -8 (maximum stability) to 50 (minimum stability). According to the manufacturer, PV were classified as “high stability” (PV between -8 and 0), “medium stability” (PV between 1 and 9) and “low stability” (PV from 10 to 50).

-Bone quality assessment

**Figure 3:**
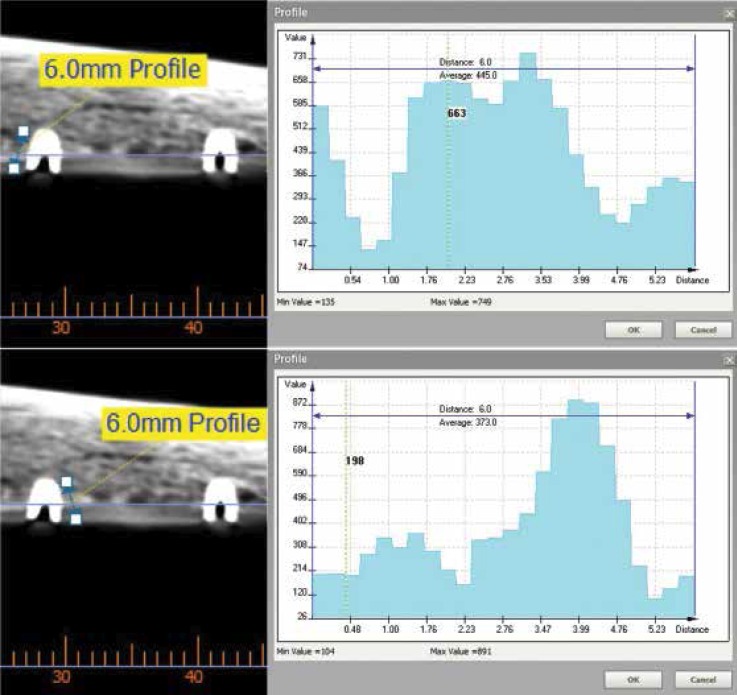
Cross-sectional images of a CT scan performed in one of the ribs using Ez3D Plus software.

Radiographic evaluation was made by a researcher blinded to the study protocol. Computed tomographies (CTs) of the ribs were taken (BrightSpeed Series CT systems, GE Healthcare, Milwaukee, WI, USA). Cross-sectional images of the ribs with 1mm in thickness were evaluated using Ez3D Plus software for Windows (Vatech Co., Korea). Bone quality was expressed in Hounsfield units (HU). HU were obtained by taking two 6mm long measurements on each side of the implant (Fig. 3). In order to eliminate measurement bias, the mean obtained was considered the value of the implant.

Misch and Kircos classification (16) was used to determine the bone quality around each implant: D1 (>1250 HU), D2 (850-1250 HU), D3 (350-850 HU) and D4 (150-350 HU). Only implants placed in D3 and D4 bone were selected in this study.

-Statistical Analysis

Statistical analysis was performed using software SPSS for Windows version 20 (SPSS Inc, Chicago, IL, USA). Means and standard deviations were obtained for ISQ, PV and bone quality (HU) of each implant. These data were assessed for a normal distribution using the Kolmogorov-Smirnov and the Shapiro-Wilk tests. As these tests exhibited that data were as stated by the theorem of the central data distribution, the one-way analysis of variance (ANOVA) was used for statistical evaluation. The results were assessed with 95% confidence intervals at a significance level of *P*<0.05.

## Results

No mobility was observed after implant placement. Ten implants were discarded because they were placed in D1 and D2 bone. Finally, 17 and 9 PHI/SHORT-I implants were placed in D3 and D4 bone, respectively. Likewise, 15 and 9 PHIA/SHORT-I implants were placed in D3 and D4 bone, respectively.

-Implant PS

In D3 bone, the mean ISQ in PHI/SHORT-I implants was 61.35 ± 4.77 and 66.43 ± 4.49 in PHIA/SHORT-I group. The mean PV was -2.76 ± 0.8 and -4.11 ± 1.24 respectively. The one-way ANOVA yielded statistically significant differences in ISQ and PV between two designs (*P*<0.005) ( Table 1).

**Table 1:**
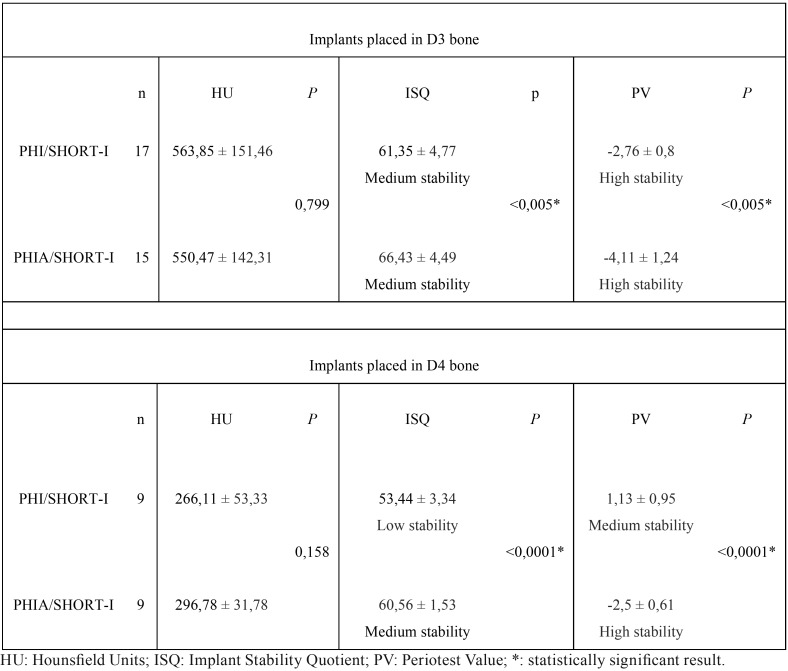
Comparison of bone quality and PS between the different implant designs studied.

In D4 bone, the mean ISQ in PHI/SHORT-I implants was 53.44 ± 3.34 and 60.56 ± 1.53 in PHIA/SHORT-I group. The mean PV was 1.13 ± 0.95 and -2.5 ± 0.61 respectively. The one-way ANOVA revealed statistically significant differences in ISQ and PV between two designs (*P*<0.005) ( Table 1).

-Bone quality

Bone quality surrounding PHI/SHORT-I implants was D3 (563.85 ± 151.46 HU) and D4 (266.11 ± 53.33 HU). The one-way ANOVA showed statistically significant difference in bone quality for the same implant group (*P*<0.005) ( Table 2).

**Table 2:**
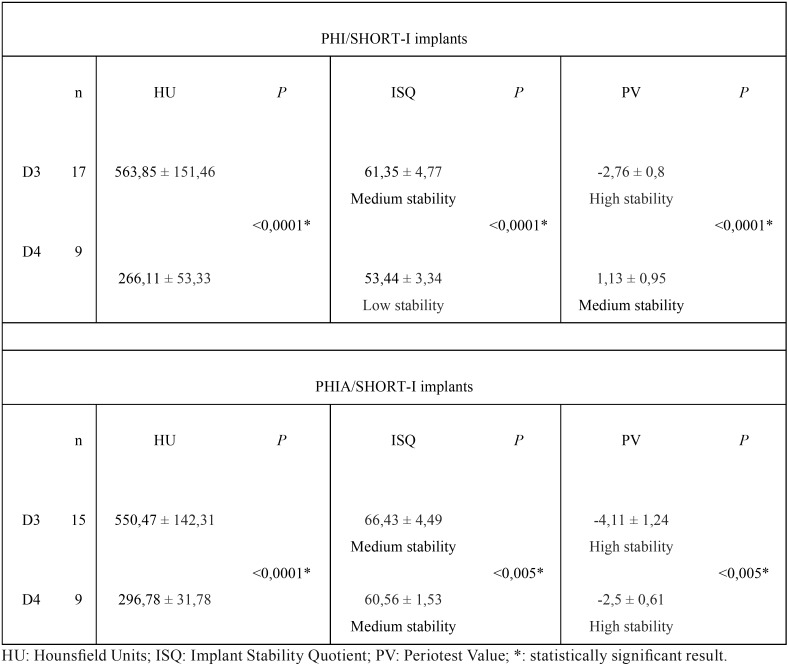
Comparison of implant designs and PS between the different bone quality observed.

Bone quality surrounding PHIA/SHORT-I implants was D3 (550.47 ± 142.31 HU) and D4 (296.78 ± 31.78 HU). The one-way ANOVA showed statistically significant differences in bone quality for the same implant group (*P*<0.005) ( Table 2).

## Discussion

In the present study, as bone tissue does not display homogenous density, bovine bone was preferred instead of polyurethane blocks that show homogeneous density (17). Nonetheless, it was therefore necessary to assess the bone quality in HU as already described (18,19).

Osstell™ and Periotest® devices were applied to assess PS. Andreotti *et al.* (20) in a systematic review concluded that there was a lack of consensus in the stability classification between these two devices. In this study occurs in a similar manner, where no agreement was found. ISQ and PV of PHIA/SHORT-I implants placed in D3 bone were 66.43 ± 4.49 (medium stability) and -4.11 ± 1.24 (high stability), respectively. In D4 bone, ISQ and PV of PHIA/SHORT-I implants were 60.56 ± 1.53 (medium stability) and -2.5 ± 0.61 (high stability), respectively. This lack of consensus affects in the loading protocols depending on the device applied in the procedure. ISQ values higher than 65 would allow us to perform an early loading (21), while ISQ values between 60 and 65 would allow for conventional loading (22). Meanwhile, PV between -8 and 0 indicated that the implant loading could be performed. Thus, the same device should be always used in follow-up analyses and clinical and radiographic examinations should also be performed (20).

Reich *et al.* (23) reported 66.9 ± 8.9 ISQ in 5-7mm long implants placed in D3 and D4 bone of the maxilla. These results are similar to those obtained in D3 bone for PHIA/SHORT-I (66.43 ± 4.49 ISQ) short implants of our study. Alonso *et al.* (24), in a prospective cohort study obtained that 6mm long implants showed higher PS in D3 bone than in D4 bone. They achieved 69,72 ± 4,35 ISQ in D3 bone and 63,68 ± 8,79 ISQ in D4 bone. This difference is similar to the one obtained in our study with PHIA/SHORT-I implants, where 66.43 ± 4.49 ISQ and 60.56 ± 1.53 ISQ were achieved in D3 and D4 bone, respectively. However, Alonso *et al.* (24) study classified the bone type based on the clinician tactile perception, while in our study it was assessed based on HU according to Misch and Kircos classification (16).

In our study, statistically significant differences were observed between D3 and D4 bone in both implant groups, which was also reflected in statistically higher PS in implants placed in D3 bone. Moreover, when the same bone quality was compared, double thread design implants obtained statistically higher differences in comparison with single thread design group. These findings concur with the scientific literature that states that an increase in bone quality also increases the PS of the implants (24-26), and that implant design is another important factor for obtaining PS (27,28).

The differences between thread designs of the implants studied may have been the main reason for these results. Our study reported that double thread design short implants obtained higher PS in D3 and D4 bone, which would lead to a greater success rate of short implants according to Griffin and Cheung (29). This also would avoid part of the complications of short implants, which mostly happen in the preprosthetic period (30).

In conclusion, this in vitro study showed that short implants geometry with double thread obtained higher primary stability with Osstell™ and Periotest® devices in comparison with single thread short implants in low quality bone. Nonetheless, further clinical studies are needed to validate these results.
